# A literature review of non-financial conflicts of interest in healthcare research and publication

**DOI:** 10.1186/s12910-025-01221-5

**Published:** 2025-05-15

**Authors:** David Bauer, Devin A. Orchard, Philip G. Day, Marc Tunzi, David J. Satin

**Affiliations:** 1 University of Minnesota Medical School, 420 Delaware St SE, Minneapolis, MN 55455 USA; 2 Department of Family Medicine and Community Health, University of Massachusetts Chan Medical School, Worcester, MA USA; 3 Family Medicine Residency, Natividad Medical Center, 1441 Constitution Boulevard, Salinas, CA 93906 USA; 4 Department of Family and Community Medicine, University of California, San Francisco, San Francisco, CA USA; 5 Department of Family Medicine and Community Health, Center for Bioethics, University of Minnesota Medical School, Minneapolis, MN USA

**Keywords:** Conflict of interest, Ethics, Publication, Research, Non-financial, Intellectual, Personal, Institutional

## Abstract

**Background:**

Conflicts of interest (COIs) in healthcare research have received substantial attention over the past three decades. Although financial COI (FCOI) has an extensive literature, publications about non-financial COI (NFCOI) are comparatively rare. Disagreements surrounding the importance of NFCOIs in research and publication, including whether competing non-financial interests should even be considered COIs, present significant gaps in the literature. This lack of clarity prompted our literature review’s aim to determine the current consensus about how NFCOIs should be treated in healthcare research and publication.

**Methods:**

We searched the PubMed database using MeSH terms and keywords to identify articles published before November 6, 2023 about NFCOI in biomedical research and publication. We applied relevance, appropriateness, transparency, and soundness (RATS) criteria to develop a final dataset of 206 publications and reported using the Preferred Reporting Items for Systematic Reviews and Meta-Analyses (PRISMA) flow diagram. Qualitative and quantitative analyses revealed major themes and conclusions regarding consensus within the field.

**Results:**

The literature centers around fundamental disagreements about (1) whether competing non-financial interests constitute COIs like FCOIs, (2) whether they need to be addressed in research, and (3) whether they should be managed with disclosure or with other strategies. Despite these disagreements, the balance of evidence and arguments suggests that (1) NFCOIs are meaningful conceptual entities like FCOIs [96%], (2) they require management [76%], and (3) disclosure is necessary but insufficient [55%] or necessary and sufficient [27%] as a management strategy.

**Conclusion:**

The topic of NFCOI enjoys far less attention and consensus compared to FCOI’s robust body of literature developed over decades. We found general agreement about the relevance of NFCOIs and the need to address them, but not how to do so. Our results are consistent with Wiersma et al., the first review on this topic. Taken together, these reviews suggest a path forward for researchers, publishers, and healthcare professionals requiring new approaches for NFCOI management.

## Introduction

**Fig. 1:**
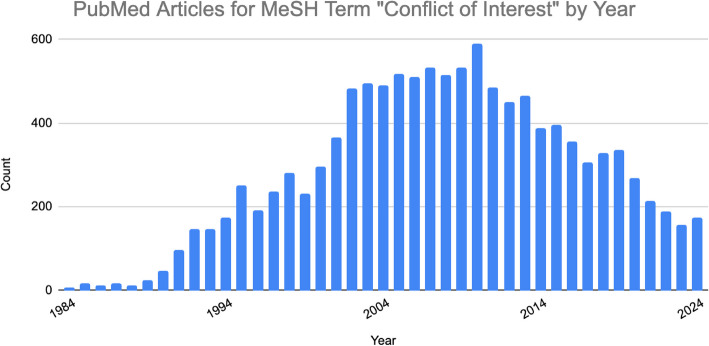
Number of PubMed articles found using the search term "Conflict of Interest"[MeSH] by year

Conflict of interest (COI) has been a prominent topic in the medical literature for over three decades (Fig. 1).

Commonly understood as a situation in which an individual’s secondary interest might generate a risk to their ability to fulfill their duties related to a primary interest [1], COI management has clear implications for the integrity of medical research and practice. Although a robust body of literature has developed around the topic, it has focused primarily on financial conflicts of interest (FCOIs) such as the risk of bias that is introduced when industry sources financially support research related to their products or give gifts of monetary value to researchers.

Non-financial conflicts of interest (NFCOIs) have received comparatively little attention [2–4]. NFCOIs are factors unrelated to financial interests that might compromise an individual’s ability to properly carry out their duties related to research or publication. They can take the form of academic, intellectual, personal, or political conflicts. A common example might be an unblinded reviewer being a close friend or staunch rival of a submitting author. A more controversial example might be an author holding a leadership position in an advocacy organization whose cause directly relates to the research at hand. Although personal relationships and ideological commitments are more difficult to measure than financial payments, the potential for conflicts to compromise the veracity of our literature is still purported to be at stake.

A recent example resulted in the retraction of three articles from Sage journals due in part to author affiliations with advocacy organizations. The lead author and multiple co-authors of articles discussing different methods of abortion had connections with pro-life organizations despite a declaration of no conflict of interest. Post publication peer-review determined that the articles were methodologically flawed, relied upon faulty assumptions, contained errors in data analysis, and represented data in a misleading manner [5]. 

An initial analysis of the state of the literature might suggest that the problem of NFCOI is already solved since it is recognized by well-respected international organizations such as the Committee on Public Ethics (COPE), the International Committee of Medical Journal Editors (ICMJE), and the World Association of Medical Editors (WAME). These entities even provide guidelines that address how NFCOIs should be handled in the course of peer review, editing, and publishing in academic journals [6–8]. However, discord persists, as adherence to these guidelines is not uniform, and there is no guarantee that those who agree with them are consistent in their application of the principles described within. Furthermore, there is no mechanism to confirm or otherwise enforce adherence to these guidelines.

Disagreements surrounding the importance of NFCOIs in research and how they should be managed are serious concerns. On the one hand, if NFCOIs are meaningful conceptual entities, like FCOIs, they have the potential to bias the evidence base. If NFCOIs of researchers, reviewers, or editors result in the dissemination of manuscripts containing misinformation or disinformation, time and resources will be spent attempting to replicate those studies. The biased data or analysis will inaccurately inform the actions of clinicians, policymakers, and advocacy groups. If discovered, public trust will be shaken by the failure to properly manage the NFCOIs. This cascade undermines the integrity of the scientific enterprise, exacerbates the public’s mistrust of scientific information, and reduces the quality of patient care.

On the other hand, if NFCOIs are conceptually incoherent, or simply not COIs warranting a response similar to financial conflicts, resources should not be spent tracking and managing them. The aforementioned biased information and public mistrust would be misplaced. Worse, our biomedical institutions’ mistaken interpretation of NFCOIs as real threats to scientific integrity will have created an erroneous cascade. NFCOI’s contribution to the skepticism of clinicians and the public would indeed be a monster of our own creation. Calls for such different courses of action make resolving this point of contention worthwhile. The lack of unanimity in practically managing or disregarding NFCOIs in healthcare research and publication prompted this literature review aiming to determine the current consensus about how NFCOIs should be treated.

We conducted this work with the intention and understanding that we were creating the first review of this topic. However, upon completion of our data extraction, synthesis, and initial submission for publication, we learned that the first review of this topic had been independently conducted by Wiersma et al. [9] during a similar time frame and had been accepted for publication, though not yet published. Neither team had knowledge of the other team when they conducted their research. Following publication of Wiersma et al. we learned that their study yielded strikingly similar findings, even identifying and focusing on similar questions debated in the literature. Given the similarities, we have elected to add a comparison of the two reviews in our discussion section. Our review serves as an independent complementary study, presenting a more robust finding for a field that, until now, has not had a clear voice.

## Methods

**Fig. 2:**
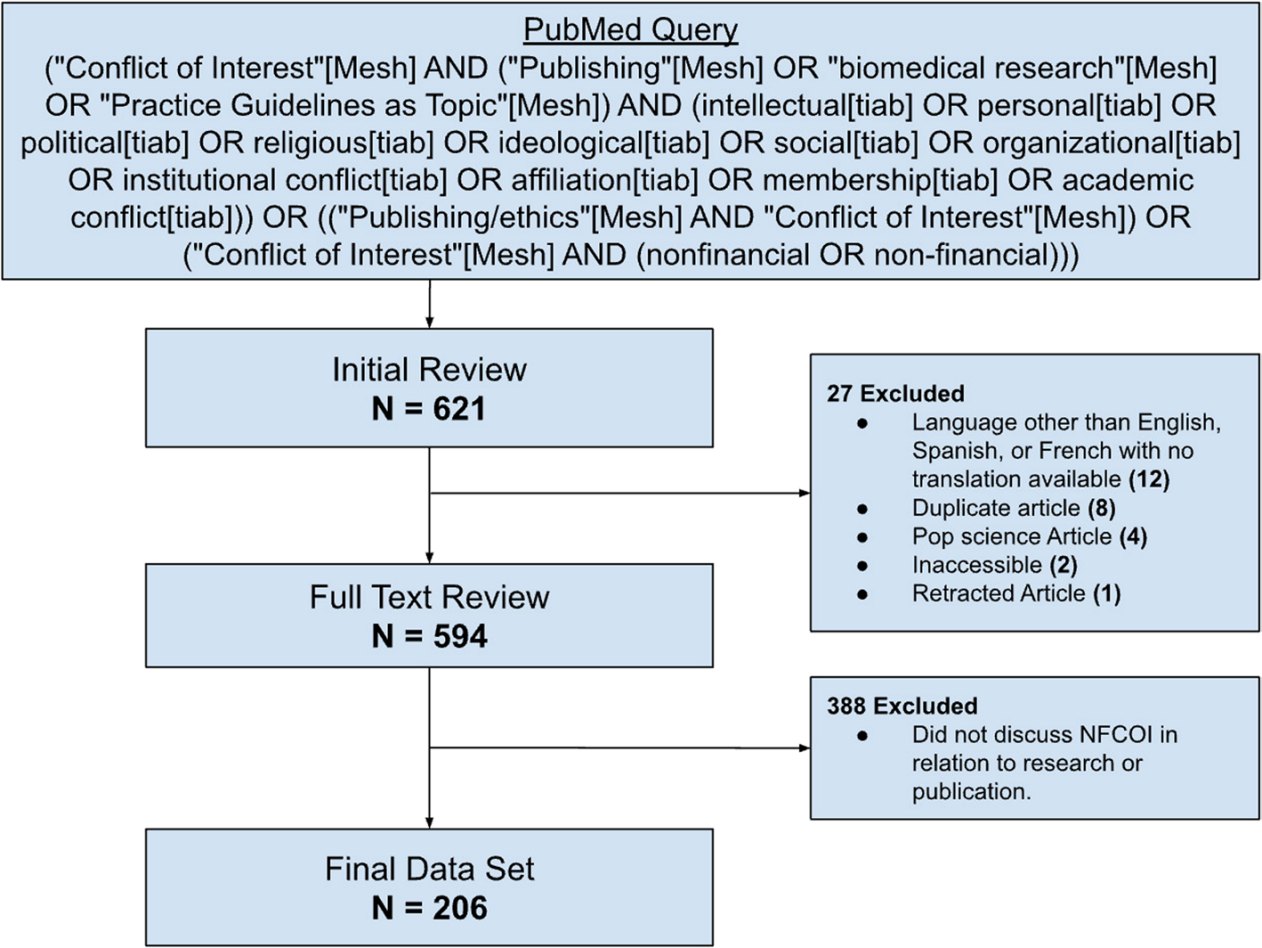
PRISMA flow diagram of literature search reporting strategy. Abbreviated terms: [tiab] indicates title and abstract search. [MeSH] indicates Medical Subject Heading search

The PRISMA flow diagram was followed for reporting purposes [10]. We reviewed PubMed for healthcare literature relevant to the topic of NFCOI focused on research and publication before November 6, 2023. The search string is noted in Fig. 2.

### Inclusion and exclusion criteria

Articles were included if they discussed NFCOI in general or a specific form of NFCOI such as academic, intellectual, personal, or political conflicts, in the context of research and publication. Articles were excluded if they did not specifically discuss NFCOI in research or publication; they were in a language other than English, Spanish, or French (the languages fluently spoken among our authors); they were reprints of the same article with a different title or in different journals; they were inaccessible to all members of the research team; they had been retracted; or they were non-academic writings such as popular science articles.

### Screening and selection of articles

Two reviewers (DAB, DAO) independently evaluated the full dataset (*N* = 621) for inclusion and exclusion. Disagreements between the two primary reviewers about an article’s inclusion were then settled by the independent evaluation of a third reviewer (DJS). Initial review (*N* = 621) resulted in the exclusion of 27 articles for article form (language, reprint, accessibility…). Full-text review of the remaining 594 articles eliminated 388 for article content (no discussion of NFCOI in research or publication), resulting in a final data set of 206 (Fig. 2).

Two reviewers (DAB, DAO) then independently coded each article in the final dataset of 206 according to article type and content, with disagreements resolved by a third reviewer (DJS). Our content categorization arose from iterative rounds of reading the existing literature and identifying themes. The following themes were identified concerning NFCOIs in research and publication:

1. Whether competing non-financial interests constitute COIs like FCOIs
2. Whether NFCOIs require management
3. Whether NFCOIs should be managed with disclosure or with other strategies

## Results

### Article type

Articles were categorized by article type and content as shown in Tables 1 and 2.

**Table 1:** Analysis of articles according to article type

Article Type	Articles (percent of total)	Articles that say NFCOI is a COI (percent of article type)	Articles that say NFCOI is not a COI (percent of article type)
Editorial/Commentary	85 (41.26%)^a^	82 (96.47%)^a^	4 (4.71%)^a^
Full Length Analysis	53 (25.73%)	50 (94.34%)	3 (5.66%)
Quantitative analysis of journal policies	18 (8.74%)	17 (94.44%)	1 (5.56%)
Quantitative analysis of journal policy adherence	18 (8.74%)	18 (100%)	0 (0%)
Quantitative Analysis of policies and adherence	7 (3.40%)	7 (100%)	0 (0%)
Review Article	10 (4.85%	10 (100%)	0 (0%)
Letter	7 (3.40%)	7 (100%)	0 (0%)
Guideline	4 (1.94%)	4 (100%)	0 (0%)
Research support	2 (0.97%)	2 (100%)	0 (0%)
Special Statement	1 (0.49%)	1 (100%)	0 (0%)
Symposium	1 (0.49%)	1 (100%)	0 (0%)

^a^The sum of the numerators is one greater than the denominator because one article contained arguments both for and against

**Table 2:** Analysis of articles according to article content

Article Content	Articles (Percent)
Argues that NFCOIs are COIs	199/206 (96.12%)^a^
Argues that NFCOIs should not be considered COIs	8/206 (3.40%)^a^
NFCOIs are important and need management	151/206 (72.82%)^a^
Treats disclosure of NFCOIs as insufficient	84/150 (55.33%)
Treats disclosure of NFCOIs as sufficient	41/150 (27.33%)
Does not directly discuss disclosure or other management strategies	26/150 (17.33%)
No statements on the importance of managing NFCOIs	47/206 (22.82%)
NFCOIs should not be managed similarly to FCOIs	9/206 (3.88%)^a^
Mentions that disclosure of NFCOI has significant drawbacks	30/206 (14.56%)
Mentions that disclosure of NFCOIs distracts from FCOIs	6/206 (2.91%)
Discussed instances of NFCOI in research or publication	6/206 (2.91%)
Contain policy statements on journal standards	42/206 (20.39%)
Argues that NFCOIs are difficult to manage as compared to FCOIs	44/206 (21.36%)
Argues that NFCOIs are neglected relative to FCOIs	46/206 (22.33%)
Discusses Author NFCOIs	179/206 (86.89%)
Discusses Reviewer NFCOIs	82/206 (39.81%)
Discusses Editor NFCOIs	79/206 (38.35%)

^a^One article contained arguments on both sides of multiple debates, resulting in one more numerator across several categories

When viewed by article type, 45% (93/206) were editorials, opinion pieces, special statements, letters, and commentaries. Full-length analyses, review articles, and research support made up 32% (65/206) of the total. Quantitative analyses made up 21% (43/206) of articles. Guidelines (2%, 4/206) or symposia (0.5%, 1/206) were rare. 19% of these articles also contained policy statements related to journal values (40/206). Discussion of occurrences of NFCOI at different levels of the research process were included in 3% (6/206) of articles.

The quantitative analyses split broadly into two categories: articles assessing the *presence* of COI policies in journals (42%, 18/43), and those assessing the *adherence* to COI policies (42%, 18/43). Seven articles (16%, 7/43) addressed both the presence of COI policy and adherence to COI policy. Those assessing the presence of COI policies endorsed the idea that journals are increasingly taking measures to manage NFCOIs, but are still doing so less commonly than they do with FCOIs [2, 11–16]. Articles assessing the adherence to COI disclosure policies found that NFCOI disclosures are happening in some cases, but are often underreported [14, 17–21] and are happening generally less than FCOI disclosures. [22, 23]

### Article content

1. Do competing non-financial interests constitute COIs like FCOIs?

Of the articles included in our sample, 96% (198/206) indicated support for the idea that competing non-financial interests should be considered COIs, 3% (7/206) argued they should not, and 0.5% (1/206) took no position.

Multiple forms of NFCOIs were proposed by articles supporting NFCOIs as COIs including: ideological conflicts, intellectual passion or dedication to a particular school of thought [2, 11, 24–40]; personal relationships including rivalry, cronyism, and animosity [2–4, 11, 12, 24–28, 30, 32, 34, 37, 39, 41–48]; academic or professional competition [11, 12, 30, 39, 41, 43, 47, 49–52]; political views, comments, and affiliations [2, 3, 11, 25, 28, 33–35, 38, 42–44, 53–64]; religious views [2, 3, 11, 25, 28, 35, 38, 42–44]; career advancement/interests [4, 29, 34–36, 45, 49, 50, 65, 66]; and conflicts based on personal reputation including glory, fame, and prestige seeking [4, 34, 50, 65–69]. Less commonly cited sources of conflict were professional relationships [3, 18, 41, 70], strong negative or positive attitude or bias towards an answer to a scientific question [41, 51, 71], previous public statements on the topic of research [18, 36, 72], individual [44, 73] and institutional [73] goals for accomplishment, organizational/institutional affiliations [18, 28, 36, 45, 74], and ties to advocacy groups [36, 38, 72, 75, 76].

Articles citing sources of NFCOIs frequently noted that greater expertise is typically accompanied by NFCOIs. Experts with the most time spent working in a given field are the most likely to have strongly held views that are not easily relinquished [77], and also a high number of social and occupational connections. This is problematic for building unbiased teams, as the relationship between NFCOI and expertise means that those with the lowest amount of relevant NFCOIs are also likely to know the least about the subject at hand [78, 79].

2. Do NFCOIs in research require management?

Of the 198/206 articles that endorsed the idea that NFCOIs are COIs, 76% (150/198) treated it as an impactful issue that required management, 24% (47/198) made no indication about its importance or expressed ambivalence, and 0.5% (1/198) made statements indicating that NFCOIs should not be addressed in research with the same intensity as FCOIs. Among the 7/206 articles that argued competing non-financial interests should not be understood as COIs, 100% (7/7) also argued that they should not be managed as COIs. The single article (1/206) that took no position on the status of NFCOIs contained arguments both for and against the importance of addressing NFCOIs.

A recurring argument against managing NFCOIs was that doing so would distract from the more pressing concern of FCOIs. Even articles supporting the management of NFCOIs acknowledged this as a possible limitation [79, 80]. A recurring theme supporting the seriousness of NFCOIs warned of the perils of neglecting NFCOIs as compared to FCOIs. Many noted the relative challenge of identifying, quantifying, and managing NFCOIs.

3. Should NFCOIs be managed with disclosure or with other strategies?

Of the 150/206 articles arguing for NFCOIs as COIs warranting management, 55% (83/150) discussed strategies in addition to disclosure, 27% (41/150) discussed disclosure alone or endorsed it as the main strategy, and the remaining 17% (26/150) did not discuss management recommendations. Across all of these categories, 13% (20/150) also included arguments either discouraging disclosure as a management strategy or encouraging caution with its use. These articles argued that disclosure comes with serious drawbacks, including distracting attention from FCOI [79, 80], invasion of privacy [24, 72], and the creation of a “conflict confessional” where biasing factors are forgiven [59].

The most commonly suggested methods for NFCOI management, aside from disclosure, included promoting a culture of skepticism [81–83], recusal of reviewers [39, 43, 81] or editors [43, 49, 50, 81, 84–86] when significant NFCOIs are present, open peer review [27, 31, 87], and double-blinded peer-review [18, 80, 87, 88]. Less discussed strategies included “continued dedication to the scientific method” [89], inviting editorials rigorously critiquing articles with COIs [90], and submitting protocols before results have been obtained [38]. One article also called for the implementation of lessons from the field of unconscious bias research, with “focused training, personal awareness, and faculty role modeling” [73].

## Discussion

1. Do competing non-financial interests constitute COIs like FCOIs?

The vast majority of authors in our review consider competing non-financial interests to be COIs. Many articles named different forms of competing non-financial interests and assumed they could bias an individual involved in the research or publication process. However, several explanations were given for the categorization of NFCOIs as COIs, including: NFCOIs might introduce bias which skews interpretation of the data to be published [91]; patient and public perception of COIs can be as important as the actual presence of COI [48, 60, 89, 92–96]; NFCOIs might encourage an individual to perform research in a hasty, faulty, or dishonest manner to reach publication as quickly as possible [68]; NFCOIs might result in “spin,” influencing a researcher to use the analyses most likely to produce desired results [68, 97], include only those results aligning with their view, and/or downplay the limitations of their work [97]; author affiliation influences what is emphasized when data is reported [98]; and empirical data shows that disparate forms of NFCOIs can lead to bias for authors [3, 35, 98–101], reviewers [3, 84, 101], and others involved in the publication process [102]. Despite these specific concerns, an overarching theme across many authors is that NFCOIs are more difficult to define and track than FCOIs. This may partially explain their relative neglect in medical ethical discourse.

Two common themes arose in the articles arguing against the conception of competing non-financial interests as COIs. The first is that such interests are intrinsic to research and impossible to control or eliminate [103, 104]. The second is that the definition of NFCOI is so broad and nonspecific that it loses any intelligible meaning [105, 106] or just becomes another phrase for bias [104]. Additionally, individual articles made arguments that the direction of bias produced by supposed NFCOIs is inconsistent [103], they only affect discrete situations [103], and there is a lack of empirical studies confirming their impact on research [105].

A point raised by articles both for and against considering competing non-financial interests as COIs is the lack of practical, objective criteria by which one would determine when a competing interest rises to the level of a COI. Most articles that discussed a “cutoff” for NFCOIs argued for some form of a “reasonable person” standard, whereby competing non-financial interests deserve attention if they might make a reasonable person doubt the integrity of an academic work, or if they might embarrass a person if they were revealed after publication rather than disclosed beforehand [58, 74, 83, 94].

One article offered an approach to combat the potential broadness and subjectivity of NFCOI declarations, arguing the need to disclose should be based on the relevance of competing interests as determined by the criteria of pertinence, substantiality, and immutability [44]. For example, although religion can fall into the category of NFCOI, it need not be declared unless it is pertinent, substantial, and immutable. In the case of medical assistance in dying, religious views may be pertinent, may result in substantially different outcomes, and may be immutable to change, and thus should be declared. Others offered a spectrum-model of NFCOI. Within this framework, the goal is to have the fewest NFCOIs in a given project, accepting that zero NFCOIs is impossible [107].

2. Do NFCOIs in research require management?

A large majority of papers argued that NFCOIs are of equal moral relevance as FCOIs, and must be managed accordingly. Some argued that NFCOIs are more influential than FCOIs [80]. Justifications for this stance included that: NFCOIs reduce the trustworthiness of a given study [83]; the bias that NFCOIs introduce can impact “grant awards and renewals, appointment to positions, promotion, and tenure;” [58] society’s trust in the scientific enterprise to some degree hinges upon the management of such ethical issues [48]; NFCOIs lead to unethical behaviors including selective publication (aka “salami slicing”), duplicate publication, plagiarism, and digital image enhancement [108]; and the bias NFCOIs may cause in primary studies [109] and systematic reviews [109, 110] undermines the work of policymakers [22, 110], advocacy groups, patients [110], physicians [97, 110, 111], and the legal system [97] to the degree that they rely on this evidence.

Those pushing back against the idea of addressing NFCOIs in the same manner as FCOIs offered practical and logical reasons. As detailed in the results, many noted that NFCOIs might divert attention away from the more significant concern of FCOI [78, 103, 104, 112], thereby reducing our ability to address the tangible and consequential threats posed by FCOI. One argument hinged on the meaningfully different impacts of NFCOIs and FCOIs, as NFCOIs “may provide reasons to suspect cognitive bias but they do not typically involve a loss of trust in a social role. The same cannot be said for [FCOIs.]” [112] Another line of argumentation implied that attempts to manage NFCOIs might lead to exclusion of individuals with varied perspectives [10–106, 113], thus hurting the quality of science. Finally, several authors agreed that NFCOIs are properly termed COIs but warrant no management, explicitly stating that FCOIs are of higher ethical priority [89, 112].

3. Should NFCOIs be managed with disclosure or with other strategies?

This was the most heterogeneous result with a narrow majority endorsing management strategies in addition to disclosure. Debate about the role of disclosure ran the full logical spectrum. Some authors treated disclosure as sufficient, arguing that it would provide readers the information necessary to assess the validity of the claims within an academic work [58, 93]. Others argued that, although lackluster, disclosure is the best (or only) realistic option [114]. Most argued that disclosure is necessary but insufficient to address NFCOIs [115]. A small minority argued that disclosure may be *inappropriate,* and has significant drawbacks that should make us hesitant to rely on it as a strategy for NFCOI management [112, 116–121].

The most common argument in favor of disclosure as a management strategy for NFCOIs was that it gives readers the information necessary to evaluate the merits of the research for themselves [58, 91, 93, 122]. In the “necessary but not sufficient” camp, some argued that disclosure allows editors and reviewers to determine whether an author is too conflicted and must be recused or even rejected [42, 59]. Beyond these examples, explanations were rarely given by authors endorsing disclosure, treating it as the de facto management strategy.

Those who did not endorse disclosure as sufficient offered several distinct arguments. First, as a direct counter to disclosure giving readers the information they need to evaluate the research, many articles argued that readers do not necessarily use disclosures to weigh the biases of authors as effectively as we might believe [112, 123]. Additionally, disclosure of certain types of NFCOI might result in invasion of privacy [24, 105], for example with regards to conflicts involving religious affiliations, sexual orientation, gender identity, disability status, or illnesses suffered by members of one’s family. Articles posited that disclosure can be an empty proclamation, doing nothing to address the underlying bias that concerns us [97, 115]. Worse, disclosure may have a moral licensing effect [116–119], whereby individuals feel less inclined to check their own biases once they have made a declaration. Additionally, the “noise” generated by including disclosure of NFCOIs might drown out FCOI disclosures [115].

Many authors discussed the challenges of enforcing disclosure policies. Policies that do exist are often poorly defined [124]. There is little consensus on an objective benchmark for enacting these policies [123]. Moreover, disclosure policies rely on the honesty and integrity of the authors themselves [107, 125, 126], as it is not feasible for editorial staff to investigate and police all submissions. Author self-disclosure is further complicated by requiring self-awareness. COIs can be present without conscious awareness [102]. Additionally, journals often lack clear procedures to verify disclosure [12, 13] and to respond if incomplete disclosure is discovered [12, 124]. Procedures described for management of undisclosed NFCOIs discovered after publication were inconsistent: published corrections/letters [48, 127], “warnings, retractions, statement of lost confidence, notification of the author’s primary institution, and exclusion from publication in the journal for a specified time frame” [128]. One prominent article discussed “registries of interests,” but conceded that these are not feasible to create and maintain [129].

### Comparing and contrasting with Wiersma et al

Our methods were similar to Wiersma et al. [9] in that we used similar keywords and Medical Subject Headings (MeSH). Our methods differed in that we focused our search exclusively on healthcare through the PubMed database while their search strategy included articles from Embase, SCOPUS, and Web of Science in addition to PubMed. Our final dataset included 206 articles, whereas Wiersma et al. had 190, likely because we included search terms specific to the types of competing non-financial interests, such as “intellectual,” “political,” and “religious.” A final difference in search strings is that Wiersma et al.’s inclusion criteria allowed for articles that discussed NFCOI outside of research and publication, such as in medical education and practice. In sum, our search represents a deeper dive into NFCOI’s impacts on healthcare research and publication, whereas Wiersma et al.’s speaks to NFCOIs across broader contexts.

**Fig. 3:**
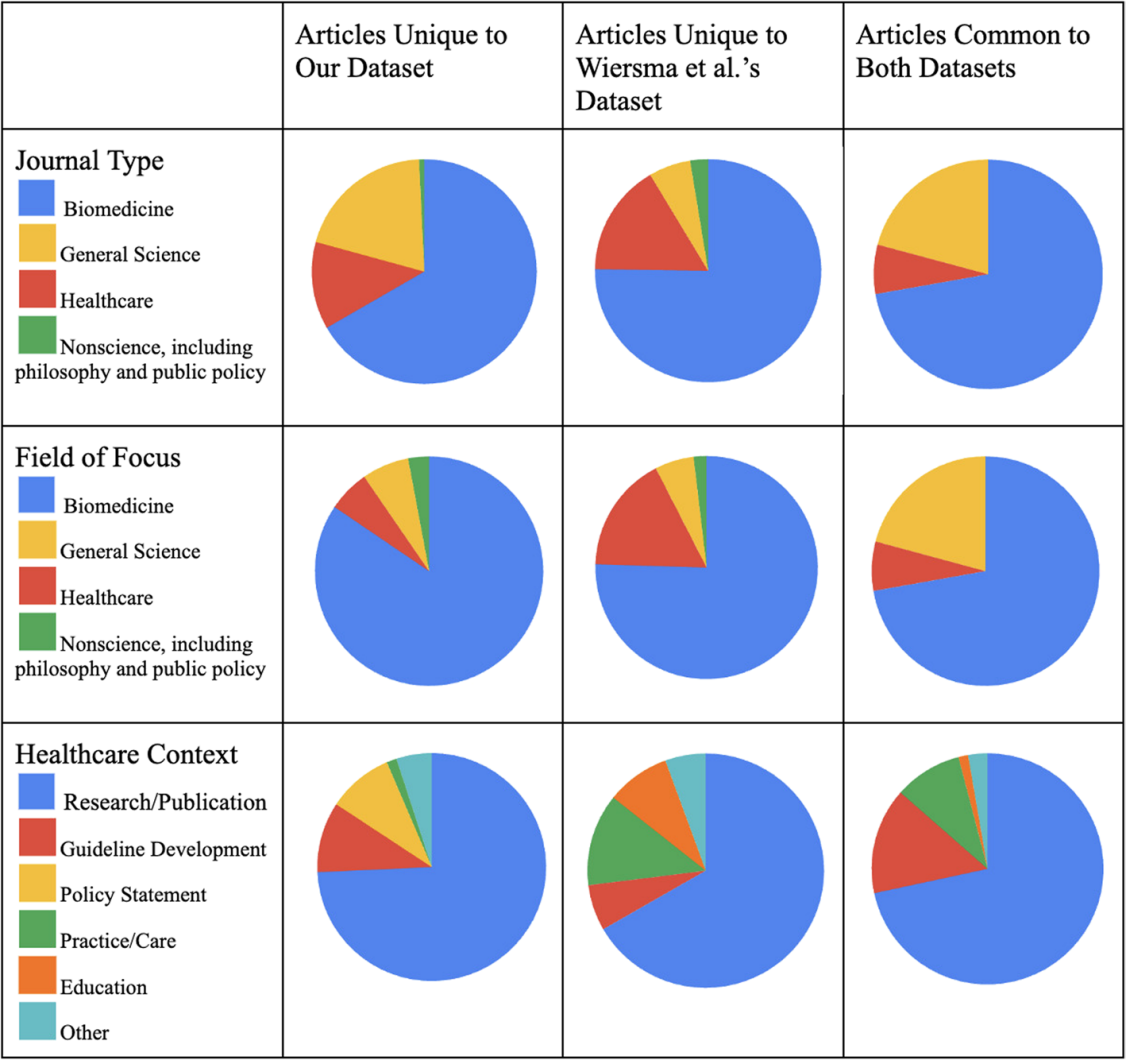
Distribution of articles and journals. The number of articles in “Field of Focus” and “Healthcare Context” exceed the total number of articles because some articles fulfilled multiple categories

Comparing our results with Wiersma et al., 72 articles were shared between the datasets, 134 were unique to ours, and 118 were unique to theirs. Despite mostly different datasets, we had similar distributions of journal types and articles’ fields of focus (biomedical, healthcare, science, and non-science). NFCOI in research and publication constituted the most common healthcare context of both datasets. Our dataset’s second and third most common healthcare contexts were policy statements and guideline development. In contrast, Wiersma et al.’s second and third most common healthcare contexts were practice and education (Fig. 3).

Remarkably, Wiersma et al.’s results and discussion were organized around a similar three core questions as our review: definition and legitimacy of NFCOIs as COIs, whether NFCOIs require management, and what that management should entail. Despite unique search strategies yielding different datasets, both our research groups came to similar conclusions for each of the three core questions.

In line with our results, Wiersma et al. found the preponderance of articles supported the conception of NFCOIs as COIs, with a small number of prominent voices arguing the contrary. They identified many of the same NFCOI types, including career-related, interpersonal, status related, and belief or viewpoint based.

Regarding management of NFCOIs, 73% of our total articles^1^ versus 55% of Wiersma et al.’s endorsed the need for some form of intervention. Only 4% of our total articles rejected the need for management versus 11% of Wiersma et al.’s. Arguments for and against the need for management were similar across datasets. Arguments in favor included that NFCOIs are of equal or greater importance to FCOIs, and that they disrupt research integrity. Arguments against were led by concerns that management of NFCOIs would distract from FCOIs, and that NFCOI is defined too broadly to be managed.

Although Wiersma et al. did not summarize recommended NFCOI strategies quantitatively, they acknowledged disclosure as a common strategy. Like us, they found frequent discussion that disclosure is necessary but not sufficient, and can have significant drawbacks. Alternative strategies identified by both our studies include open discussion, reflexivity, management according to severity, use of scientific methods, balancing competing interests, registries, and policies.

### Strengths and limitations

This review has three unique strengths. First, it independently corroborates the findings of Wiersma [9] et al., having independently conducted the literature search, data analysis, and conclusions prior to the publication of their article. Moreover, two reviewers independently read and coded each article at the 621 and 206 stages, with a third, senior author adjudicating disagreements. Taken together, Wiersma et al. and our study produced similar results from complementary datasets. Second, this review quantifies the number of articles including arguments for and against our three major questions. Third, Wiersma et al. cite their own NFCOI as a limitation given their history of argumentation towards their review’s conclusions. Without such NFCOIs of our own on this topic, our study even more strongly supports Wiersma et al.’s conclusions that NFCOIs are a serious problem warranting management.

We identify several limitations. First, we limited our search to the PubMed database focusing on research and publication. This resulted in a deep dive into NFCOI in healthcare research and publication. Although this was an intentional choice, additional databases may have revealed additional articles and entire fields with differing perspectives. Acknowledging this, Wiersma et al.’s similar findings with the use of multiple databases makes this unlikely.

Second, our study is limited by a search strategy that could be biased for articles favoring the view that competing non-financial interests are COIs requiring management. In searching the literature for COIs that are non-financial, articles that do not even include the term COI would have been missed. This could be one explanation for our results heavily favoring treating NFCOIs as COIs requiring management. Furthermore, our search string was designed to retrieve only articles indexed with the ‘Conflict of Interest’ MeSH term, with no ‘Conflict of Interest’ keyword included in our search string. This strategy prevented creation of an initial dataset with an overwhelming number of irrelevant articles containing the phrase ‘Conflict of Interest’ in their disclosures. However, this led to omission of relevant articles (e.g. Resnik DB) [130] that discuss NFCOI but had not been assigned the MeSH term at the time of our search.

Third, we found that many different terms refer to NFCOIs. Subtypes, such as “Intellectual COI,” “Personal COI,” and “Academic COI” are sometimes used interchangeably with the term “non-financial COI.” Although we included eleven subtypes of NFCOI, our search may have missed articles containing other variations. Nevertheless, our inclusion of subtypes may help explain our larger final dataset as compared to Wiersma et al.

## Conclusion

The topic of NFCOI enjoys far less attention and consensus compared to FCOI’s robust body of literature developed over decades. The existing literature largely agrees that competing non-financial interests constitute COIs, but there remain some prominent contrarian voices. Despite a general consensus about the relevance of NFCOIs, there is ongoing debate surrounding management. Most authors endorse disclosure as necessary but not sufficient. Further research is needed to clarify alternative management strategies. Our results are consistent with Wiersma et al., the first review on this topic. Given that our two studies were conducted independently, at the same time, and without knowledge of the other, the conclusions are more robust. Taken together, these reviews suggest that a path forward for researchers, publishers, and healthcare professionals requires new approaches to achieve greater consensus for NFCOI management. We believe such consensus will encourage greater adherence to more consistent policies.

## Data Availability

Data that support the findings of this study have been deposited in Science Data Bank with the DOI www.doi.org/10.57760/sciencedb.18058.

## Footnotes

1 For purposes of comparison, we have converted our quantitative analysis in this section to match Wiersma et al.’s methodology.
